# Heme oxygenase-1 modulates CD62E-dependent endothelial cell–monocyte interactions and mitigates HLA-I-induced transplant vasculopathy in mice

**DOI:** 10.3389/fimmu.2025.1447319

**Published:** 2025-03-07

**Authors:** Laura Schuster, Marcin Zaradzki, Henrike Janssen, Nadia Gallenstein, Melanie Etheredge, Ilse Hofmann, Markus A. Weigand, Stephan Immenschuh, Jan Larmann

**Affiliations:** ^1^ Department of Anesthesiology, Heidelberg University Hospital, Heidelberg, Germany; ^2^ Faculty of Biosciences, Heidelberg University, Heidelberg, Germany; ^3^ Department of Cardiac Surgery, Heidelberg University Hospital, Heidelberg, Germany; ^4^ Department of Anesthesiology, University Hospital Rheinisch-Westfälische Technische Hochschule (RWTH) Aachen, Aachen, Germany; ^5^ Division of Vascular Oncology and Metastasis, German Cancer Research Center (DKFZ), Heidelberg, Germany; ^6^ Department of Transfusion Medicine and Transplant Engineering, Hannover Medical School, Hannover, Germany

**Keywords:** transplantation, chronic rejection, heme oxygenase-1, anti-HLA-1 antibodies, accommodation, endothelial cells, adhesion, monocyte transmigration

## Abstract

The main risk factor for developing transplant vasculopathy (TV) after solid organ transplantation is *de-novo* production of donor-specific antibodies (DSAs) binding to endothelial cells (ECs) within the graft’s vasculature. Diverse leukocyte populations recruited into the vessel wall via activated ECs contribute to vascular inflammation. Subsequent smooth muscle cell proliferation results in intima hyperplasia, the pathophysiological correlate of TV. We demonstrated that incubating aortic EC with anti-HLA-I antibodies led to increased monocyte adhesion to and transmigration across an EC monolayer. Both occurred in a CD62E-dependent fashion and were sensitive toward the anti-inflammatory enzyme heme oxygenase (HO)-1 modulation. Using a murine heterotopic aortic transplantation model, we demonstrated that anti-MHC I antibody-induced TV is ameliorated by pharmacologically induced HO-1 and the application of anti-CD62E antibodies results in a deceleration of developing TV. HO-1 modulation is a promising therapeutic approach to prevent leukocyte recruitment and subsequent intima hyperplasia in TV and thus precludes organ failure.

## 1 Introduction

Solid organ transplantation is the treatment of choice for end-stage organ failure. Over recent decades, the 1-year survival after organ transplantation increased to over 90% due to continuously improved surgical techniques and perioperative care as well as increasingly effective immunosuppressive medication (1, 2). To date, the occurrence of chronic rejection determines long-term survival after organ transplantation. Every second patient develops transplant vasculopathy (TV) characterized by intima hyperplasia. The subsequent reduction in tissue perfusion limits organ survival. Preventive or therapeutic options are rare (3–5) and limited to monoclonal antibodies (6) or a combination of plasmaphereses and intravenous application of immunoglobulins (7). As therapeutic success is still limited, TV remains the major obstacle for long-term graft survival.

Owing to immunological differences between the recipient and donor organ, either a cellular or a humoral-driven immune reaction in which both pathways share common features is elicited. In the end, the activated immune system causes tissue injury which in turn leads to organ damage followed by graft failure (8). The main risk factor for developing TV is donor-specific antibodies (DSAs) recognizing MHC class I and II molecules on the cell surface of the donor organ (9). DSAs are associated with a two-fold increased risk for developing chronic rejection (10), and their concentration correlates with reduced survival time of the transplanted organ (11). DSA’s predominant role in TV has been proven by several mouse models where solely the application of anti-MHC I antibodies was capable of evoking TV, even in the absence of T and B cells or a functional complement system (12, 13).

TV particularly affects the blood vessels of the transplanted organ, and accordingly, one hallmark is intimal hyperplasia causing neointima formation and reduced vessel diameter (14). Dysfunctional endothelial cells (ECs) and migration of vascular smooth muscle cells (VSMCs) from the media into the intima as well as accumulation of collagen and elastin are responsible for the phenotypical alteration of transplanted organs undergoing chronic rejection (15, 16). Migrated VSMCs adopt a synthetic phenotype and directly influence the fate of ECs due to paracrine and autocrine effects of proinflammatory cytokines (17). Intima thickening occurs at an early timepoint after the onset of TV (18) and results in altered tissue perfusion (19) and impaired vasodilatation capacity (20) accompanied by reinforced stiffness of the vessel wall (16). ECs are a preferred target for DSA because their location predisposes them to interface between the recipient’s circulation and the graft and because they express all major sets of antigens. Besides complement activation by bound antibodies, the binding of anti-HLA-I antibodies to EC induces complement-independent upregulation of intracellular signaling pathways, such as NF-κB, ERK, and FGF, manifesting itself in phenotypical conversion and consequently in EC activation (21). EC activation results in the reinforced expression of adhesion receptors on the surface as well as intensified cytokine exocytosis (22). Established cytokine gradients and the availability of unbound adhesion receptors lead to the recruitment of leukocytes to the site of inflammation and to their transmigration into the vessel wall. The first loose interactions between ECs and leukocytes are predominantly mediated by integrins and selectins (23), and the resulting transmigration of leukocytes into the vessel wall is achieved through additional binding of specific receptors, such as PECAM, JAM A, and CD99 (24). If the immune system fails to resolve EC activation and the proinflammatory environment of the endothelium persists, chronic inflammatory status—and therefore TV—is established.

Meanwhile, histopathological analysis of transplanted organs has identified macrophages as the predominant cell type forming the mononuclear infiltrate (25). The implication of the amount of transmigrated monocytes and thus the extent of the constituted mononuclear infiltrate within the graft’s vessel wall bears a close negative correlation with organ function (26). A retrospective study revealed that transmigrated monocytes correlate with a serological occurrence of DSA and could be used as a diagnostic marker for early asymptomatic disease (27). This finding would allow for faster therapy initiation and thus prevent progression to fully developed TV. Patients with TV also bear a significantly higher number of transmigrated monocytes at an earlier timepoint after transplantation than patients who do not suffer from chronic rejection (28). Moreover, a cardiac transplantation model in mice showed that monocytes contribute to the progression of organ rejection, whereby systemic monocyte depletion decreases the extent of TV (29). Blocking specific adhesion receptors on the EC’s surface prevents monocyte transmigration and leads to a reduction in experimentally induced chronic rejection (30).

The presence of DSA does not inevitably lead to pathological changes in transplanted tissue. Stable organ function can be maintained over a longer period, a state referred to as accommodation (31). Accommodation is reflected in an anti-inflammatory EC phenotype that is noticeable in the increased expression of anti-apoptotic and antioxidative genes (32) when the expression of adhesion receptors simultaneously decreases (33). The consequences of induced anti-inflammatory pathways have not been fully determined, and accommodation—as well as its underlying mechanisms—has not been characterized in detail. A mouse-to-rat xenotransplantation model has provided evidence that the inducible isoform of heme oxygenase (HO)-1 mediates the main anti-inflammatory effect of the enzyme (34). HO-1 catalyzes the conversion of heme to biliverdin through the release of carbon monoxide (CO) and iron [Fe (II)]. Furthermore, targeted HO-1 induction protects against anti-HLA-I antibody-induced EC activation (22) and diminishes leukocyte adhesion (35). Reduced leukocyte adhesion is conveyed by biliverdin whereby low concentrations of CO impede the expression of proinflammatory cytokines, such as TNF-α and IL-1β (36). The heme derivate cobalt (III) protoporphyrin IX chloride (CoPPIX) has also been identified as an HO-1 expression enhancer that increases the homeostatic-relevant IL-8 in EC (37). Statins also exert anti-inflammatory effects in an HO-1-dependent fashion. Besides reducing cholesterol levels, statin treatment for patients suffering from TV results in declined neointima formation within the graft (38, 39).

Nevertheless, it is essential to understand the underlying mechanisms leading to the formation of a neointima and mononuclear infiltrate in more detail. This study demonstrates the effects of HO-1 expression on anti-HLA-I-induced adhesion and leukocyte transmigration on and across an endothelial monolayer. Moreover, our initial results suggest that HO-1 activity has a protective effect on developing TV in an allogenic aortic transplantation model in mice.

## 2 Materials and methods

### 2.1 Reagents

We used an anti-HLA-I antibody (clone: w6/32, isotype: IgG2a) from Thermo Fisher Scientific (Waltham, USA) and an isotype control from BioLegend (San Diego, USA). Recombinant human monocyte chemoattractant protein (MCP)-1 was obtained from PeproTech (Winterhude, GER), CoPPIX was from Frontier Scientific Services (Newark, USA), and CO-releasing molecule (CORM)-401 as well as Cell Tracker Green was from Sigma-Aldrich (St. Louis, USA). For statin treatment, pravastatin tablets from Novartis (Basel, CH) were used, and the pharmacological substances were dissolved in ultrapure H_2_O. Subsequently, carrier substances were removed and the solution was sterile-filtered. HO-1-specific siRNA (#L-006372-00-0005) and non-target siRNA control were purchased from Dharmacon (Lafayette, USA), and our transfection reagents, OptiMEM-Medium and Lipofectamine RNAi/Max, were from Thermo Fisher Scientific. RNeasy Micro Kit and QuantiTect Rev. Transcription Kit were obtained from Qiagen (Hilden, GER). TaqMan^®^ gene expression assays and TaqMan^®^ Fast Advanced Master Mix were purchased from Thermo Fisher Scientific. Polyclonal anti-human CD62E antibody and isotype control for blocking experiments *in vitro* were both from Abcam (Cambridge, GB) (mouse anti-human CD62E: ab18981 and mouse IgG isotype control: ab37355). For the blocking experiments, *in-vivo* mouse anti-mouse CD62E (clone: RME-1, isotype: IgG1, 148802, BioLegend, San Diego, USA) and mouse IgG1 isotype control (clone: MG1-45, 401402, BioLegend, San Diego, USA) were used.

### 2.2 Cell culture

Primary human aortic endothelial cells and a culture medium consisting of basal medium MV2 supplemented with growth factor MV 2 Kit were obtained from PromoCell (Heidelberg, GER) (40). ECs were cultured until reaching 80% confluency before splitting. For all experiments, ECs were used between passages 4 and 6. Stimulation of ECs with antibodies or HO modulators was conducted in a starvation medium consisting of basal medium and 2% FCS. The human monocytic cell line THP-1 was purchased from Life Technologies (Carlsbad, USA) and cultured in RPMI-1640 medium supplemented with 10% FCS and maintained at a density of 10^6^ cells/mL. ECs were stimulated with different concentrations of anti-HLA-I antibody (0.1 µg/mL or 1 µg/mL) or with the corresponding isotype control (1 µg/mL). Modulation of HO-1 activity in ECs was induced by stimulation with 5 µM of CoPPIX or 1 µM of statin for 24 h followed by further stimulation with the HO-1 modulator alone or in combination with 1 µg/mL of anti-HLA-I for the time period indicated. For siRNA transfection, ECs were cultured until 80% confluency and incubated with siRNA/Lipofectamine complexes in OptiMEM-Medium for 4 h. ECs were further cultivated in a culture medium, and RNA or proteome extraction was performed 24, 36, or 48 h after transfection.

### 2.3 Adhesion and transmigration assays

For adhesion assays, ECs were cultured in 12-well plates until they reached confluency and then stimulated. Shortly before the assay, 10^6^ THP-1 cells/mL were stained with 1 µg/mL of Cell Tracker Green (CTG) in Hank’s balanced salt solution (HBSS) medium for 30 min, washed, and resuspended in a binding buffer consisting of HBSS, 2 mM of Ca^2+^, and 2 mM of Mg^2+^. A concentration of 10^5^ THP-1 cells/mL was adjusted and cells were incubated for 15 min. The stimulation medium was replaced by 500 µL of binding buffer, and the plate was placed on a shaker at a speed of 20 rotations/min. Five hundred microliters of THP-1 suspension was added to every EC cultured well, and the plate was covered with aluminum foil and incubated for 30 min. The supernatant was removed and ECs were washed five times with binding buffer. In the end, ECs and adherent THP-1 were detached and washed, and the number of CTG^+^ cells out of the total cell count was analyzed with flow cytometry.

For transmigration assays, ECs were cultured in CIM plates until confluency followed by the indicated stimulation. The medium from the lower chamber was replaced by 160 µL of transmigration medium (50% starvation medium + 50% THP-1 culture medium) supplemented with 20 ng/mL of MCP-1. The medium from the upper chamber was aspirated and 50 µL of the transmigration medium was added. The CIM plate was placed in the xCELLigence system (OLS OMNI Life Science) and background measurement was begun. THP-1 cells (2.5 × 10^4^) resuspended in 100 µL of transmigration medium were added to the upper chamber, and the cell index (CI) was measured for 4 h. Readout included the area under the curve (AUC) of the CI.

For CD62E blocking experiments, ECs were incubated with 5 µg/mL of mouse anti-human CD62E antibody or the corresponding isotype control for 1 h prior to beginning the adhesion or transmigration experiment.

### 2.4 RT-qPCR

For quantification of mRNA expression analysis, ECs were cultured in 12-well plates until 80% confluency and stimulated either with antibodies alone or in combination with HO-1 modulators. Total RNA extraction was performed at different timepoints and 500 ng of total RNA was used for reverse transcription reaction. qPCR was performed with TaqMan^®^ Fast Advanced Master Mix protocol and TaqMan^®^ gene expression assays specific for specific target sequences. All steps were performed according to the manufacturer’s specifications. The following TaqMan^®^ gene expression assays were used: HMOX-1 (Hs01110250_m1), ESAM (Hs00332781_m1), PECAM-1 (Hs01065282_m1), ICAM-2 (Hs00609563_m1), CD99 (Hs00908458_m1), JAM-1 (Hs00170991_m1), JAM-3 (Hs00230289_m1), P-selectin (Hs00927900_m1), and E-selectin (Hs00174057_m1). GAPDH (Hs03929097_g1) was used as a housekeeping reference.

### 2.5 Flow cytometry

To assess the amount of CD62E expressed on the surface of ECs, cells were cultivated in 12-well plates until they reached 80% confluency followed by antibody stimulation with or without HO-1 modulation. After stimulation, the cells were washed, detached with Accutase solution (Merck KGaA, Darmstadt, Germany), and centrifuged. Afterward, cells were incubated with 1 µL of PE-coupled mouse anti-human CD62E antibody (322605, BioLegend, San Diego, USA) for 30 min at 4°C in the dark. Readout was the mean fluorescence intensity (MFI) of PE on ECs.

The presence of passively transferred donor-specific antibodies in recipient mice was confirmed using the plasma of Rag2 KO mice collected at the end of the experiment. A single cell suspension of Balb/c splenocytes (from the graft donor) was produced and incubated with 25 µL of plasma for 30 min on ice. The cells were washed and then stained with 1 µL of FITC-conjugated rat anti-mouse CD3 (100204, BioLegend, San Diego, USA) and 1 µL of PE-conjugated goat anti-mouse IgG (405307, BioLegend, San Diego, USA) for 30 min at 4°C in the dark. The readout was MFI of PE on CD3^+^ Balb/c splenocytes. All measurements were performed using FACSVerse™ Flow Cytometer and FACSuite Software Version 1.0.5.3840 I (both from BD Biosciences, Heidelberg, Germany).

### 2.6 Semiquantitative analysis of selected mouse and human cytokines

The Proteome Profiler Cytokine Arrays were used for human EC cell culture supernatants (ARY022B, Bio-Techne, Minneapolis, USA) and mouse plasma (ARY028, Bio-Techne, Minneapolis, USA) to measure the relative concentration of various cytokines. ECs were stimulated either with 1 µg/mL of anti-HLA-I antibody or IgG2a isotype control for 24 h in starvation medium. The supernatants were collected and pooled before being transferred onto the membranes. For the mouse cytokine array, plasma from the anti-MHC I antibody or isotype-treated mice was pooled and used for the assay. Both cytokine arrays were run in accordance with the manufacturer’s specifications.

The mean intensity of each spot was determined using ImageJ to evaluate the array. After subtracting the background of each membrane, all values were normalized to the reference spots on the same membrane and compared to the corresponding isotype control membrane.

### 2.7 Use of the heterotopic aortic transplantation model in mice to induce TV

Infrarenal transplantation of thoracic aorta segments into the abdominal aorta was performed using a protocol originally described by Koulack and colleagues (41). Briefly summarized, Balb/c donor mice, bearing the MHC I haplotype H2-K^d^ and purchased from Janvier-Labs (FRA), were euthanized using CO_2_, exsanguinated, and perfused using 0.9% saline. Thoracic aortas were explanted, segmented, and stored in PBS. B6.129S7-Rag2^tm1Mom^/J (Rag2 KO) mice, carrying a recombination activating gene 2 knockout mutation on a C57BL/6 background (JAX, #008449), were used as graft recipients. Due to their C57BL/6 background, these mice express the MHC I haplotype H2-K^b^ and represent a full MHC mismatch toward Balb/c mice. Rag2 KO mice are completely T- and B-cell-deficient and have a non-leaky immunodeficiency. At the age of 8 weeks, Rag2 KO mice were anesthetized using isoflurane. Following longitudinal laparotomy, the aorta was clamped and cut transversally. The graft was anastomosed in an end-to-end fashion with 11-0 nylon sutures (Prolene, 11-0, nylon black, S&T AG, Neuhaus, CHE) to the native abdominal aorta. The clamps were removed and the peritoneal and skin incisions were closed with 6-0 nylon sutures. For analgesia, 0.05 mg/kg of body weight (bw) buprenorphine was given immediately after induction and before the end of anesthesia. Analgesia was preserved with intraperitoneal (i.p.) injection of buprenorphine every 6 h for 2 days. Three days postoperatively, non-paralyzed mice were used for the experiments. Weekly i.p. application of 1.5 µg/g of bw anti-H-2K^d^ antibody (clone: SF1-1.1, isotype: IgG2a, BioLegend, San Diego, USA) induced TV in the transplanted vessel. After 30 days, the mice were euthanized, exsanguinated, and perfused with 0.9% saline to maintain vascular volume. The graft was harvested, embedded in O.C.T. medium (optimal cutting temperature), snap-frozen in liquid nitrogen, and stored at −80°C until ready for processing and histological examination.

Only male mice were used for all of the experiments, handled according to the recommendations of the Society of Laboratory Animal Science, and maintained under controlled SPF conditions. Animal experiments were approved by the Regional Council in Karlsruhe (permission number G222/19, date of approval 10/17/2019) and performed according to national legislation.

### 2.8 Modulation of HO-1 *in vivo*

To investigate the influence of HO-1 modulation on developing TV, the HO-1 modulators CoPPIX and statins were used. Additionally, the HO-1 metabolite CO was applied and CD62E blockage was tested. Simultaneous with the anti-MHC I antibody, H_2_O-solved CoPPIX was injected i.p. at a concentration of 5 µg/g of bw. Statins were dissolved in H_2_O and 40 µg/g of bw was administered daily in drinking water. CORM-401 was dissolved in DMSO in a nitrogen atmosphere and 30 µg/g of bw was given orally three times a week. Inactive CORM-401 (iCORM) was produced by incubating CORM-401 overnight at 60°C. Mouse anti-mouse CD62E antibody or isotype control was injected i.p. twice a week at the concentration of 3.5 µg/g of bw.

### 2.9 Morphometric analysis of grafts

Cross-sections of grafts at 5 µm thickness were prepared using a cryomicrotome (Leica Microsystems, Wetzlar, GER), and serial sections were H&E-stained for quantification of the neointima index (NI). Three distinct vascular compartments (lumen, intima, and media) were defined and the corresponding areas were determined. The NI was calculated based on the following formula (42):


$$NI=\frac{Intima\;area}{Luminal\;area\;+Intima\;area}\times \;100$$

The average of all NIs, calculated for every section independently, was assessed as the NI of the graft. For morphometric and immunohistochemical analyses, an Olympus BX63 microscope (Olympus Life Science Solutions, Waltham, USA) and CellSens (Olympus Life Science Solutions, Waltham, USA) were used for image capturing and analyzing, respectively. Besides the grafts, short segments of the naive aorta of Rag2 KO mice were explanted and H&E-stained.

### 2.10 Immunohistological analysis of grafts

Tissue sections were fixed with ice-cold acetone and blocked with Mouse on Mouse (M.O.M.) blocking reagent (Vector Laboratories, Burlingame, USA) for 30 min. Stainings were performed with the following antibodies: rat anti-mouse CD68 (MCA1957, Bio-Rad Laboratories, Hercules, USA) diluted 1:400 and incubated overnight at 4°C; goat anti-rat F(ab) Alexa Fluor^®^ 555 (ab21434, Abcam, Cambridge, UK) diluted 1:100 and incubated for 2 h at room temperature (RT); rabbit anti-mouse CD62E (ab2497, Abcam, Cambridge, UK) diluted 1:300 and incubated overnight at 4°C; goat anti-rabbit F(ab) Alexa Fluor^®^ 555 (A21428, Thermo Fisher Scientific, Waltham, USA) diluted 1:200 and incubated for 2 h at RT; mouse anti-vWF (CBMAB-V0158-YC, Creative Biolabs, Shirley, USA) diluted 1:50 and incubated overnight at 4°C; goat anti-mouse F(ab) Alexa Fluor^®^ 488 (A11017, Thermo Fisher Scientific, Waltham, USA) diluted 1:100 and incubated for 2 h at RT; and mouse anti-mouse SMA (A2547, Sigma-Aldrich, St. Louis, USA) diluted 1:400 and incubated overnight at 4°C. Nuclei were stained with DAPI and slides were mounted using a fluorescence mounting medium (Dako North America, Carpinteria, USA) and coverslipped. The number of macrophages was calculated in four randomly selected areas on each section and normalized, and the average was calculated. ImageJ was used for quantifying CD62E expression on either EC (vWF/CD62E) or VSMC (SMA/CD62E). The amount of double-positive EC or VSMC cells was also measured.

### 2.11 Statistical analysis

Statistical analyses were performed using GraphPad Prism (GraphPad Software, Inc.). Data were presented as mean ± standard error of the mean. All data sets were tested for outliers using the ROUT method, and the identified outliers were excluded from further analysis. Data were tested for Gaussian distribution using the Shapiro–Wilk test. The Student’s *t*-test was used to compare two different groups of normally distributed data. For three or more groups, normally distributed data were tested for significant differences of the means using one-way ANOVA comparing three or more groups. For data sets not following normal distribution, the Kruskal–Wallis test was used. Multifactorial analyses were performed using two-way ANOVA. To adjust for multiple testing, the *post-hoc* Sidak’s multiple comparison test was employed. Significant differences were assumed if *p <*0.05.

## 3 Results

### 3.1 Stimulation of human ECs with anti-HLA-I antibodies induces adhesion and transmigration of monocytes and is dependent on HO-1 modulation

**Figure 1:**
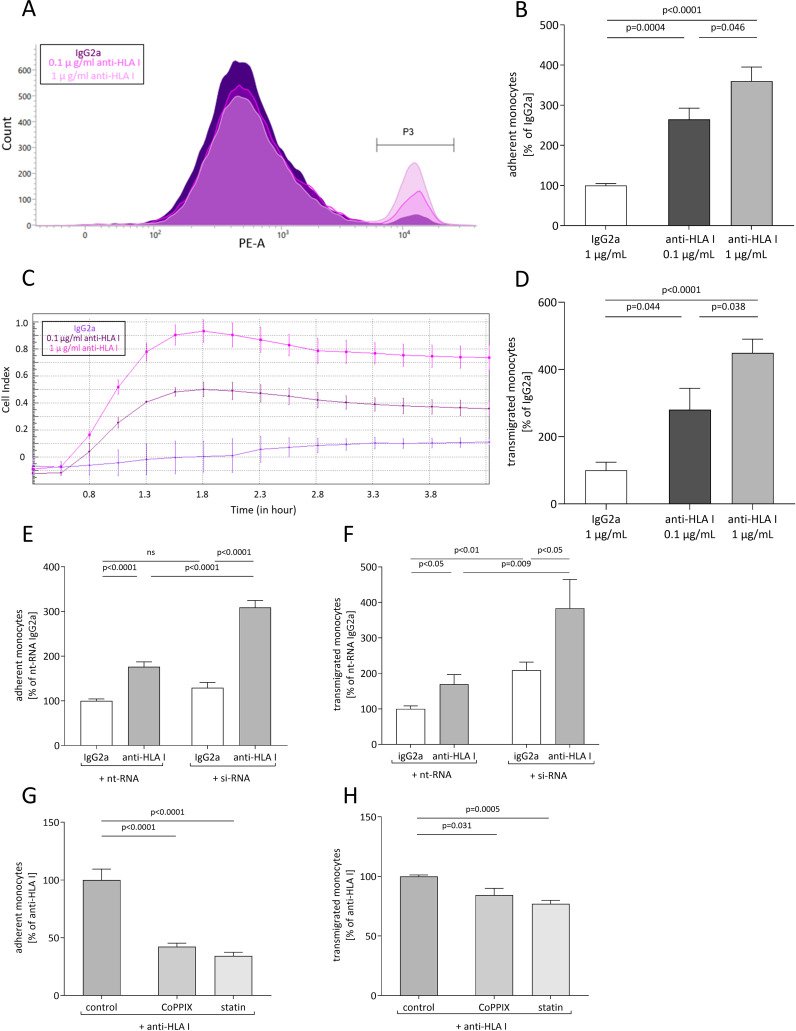
Anti-HLA-I antibody stimulation induces heme oxygenase (HO)-1-dependent adhesion and transmigration of monocytes. Human primary endothelial cells (ECs) were stimulated with 0.1 µg/mL or 1 µg/mL of anti-HLA-I antibodies or isotype control (IgG2a) in a starvation medium for 24 h. Adhesion and transmigration of THP-1 on or across an endothelial monolayer were quantified using flow cytometry and impedance measurement, respectively. **(A)** Representative flow cytometry graph for adhesion of THP-1 on stimulated ECs. Adherent THP-1 are shown in gate P3. **(B)** Statistical analysis of flow cytometry data normalized to IgG2a-treated ECs. **(C)** Representative image of impedance measurement of THP-1 transmigrated across a stimulated endothelial monolayer. **(D)** Statistical analysis of impedance data normalized to IgG2a-treated ECs. **(E, F)** HO-1 expression in ECs was suppressed with HO-1-specific siRNA transfection 36 h before anti-HLA-I antibody or IgG2a stimulation. Non-target siRNA (nt-RNA) was used as a control. Adhesion and transmigration of THP-1 were normalized to nt-RNA-transfected and IgG2a-stimulated ECs. **(G, H)** ECs were incubated with CoPPIX or statins for 48 h to increase HO-1 activity. The last 24 h of incubation occurred in the presence of anti-HLA-I antibody stimulation. Adhesion and transmigration were normalized to ECs stimulated with anti-HLA-I antibodies only. Normally distributed data (mean ± SEM) were analyzed using a one- or two-way ANOVA followed by Sidak’s multiple comparison test to adjust for multiple testing. Differences were considered statistically significant if *p <*0.05; *n* = 9.

To examine whether anti-HLA-I antibodies affect EC/monocyte interactions, confluent monolayers of human primary EC were treated either with 0.1 µg/mL or 1 µg/mL of murine pan-HLA-I antibody (w6/32) or with 1 µg/mL of isotype control (IgG2a). After 24 h of stimulation, adhesion and transmigration assays were performed. For the adhesion assays, relative numbers of fluorescent THP-1 monocytes were counted using flow cytometry after adhesion to ECs was allowed for 30 min on a rocking plate (**Figure 1A**). Stimulation with the anti-HLA-I antibody increased the adhesion of monocytes to 2.65-fold (0.1 µg/mL of anti-HLA-I antibody) and 3.60-fold (1 µg/mL of anti-HLA-I antibody) compared to the IgG2a antibody control-treated EC (**Figure 1B**). The amount of transmigrated THP-1 was quantified in a commercially available assay by impedance measurement (**Figure 1C**). In accordance with adhesion, transmigration increased up to 2.80-fold (0.1 µg/mL of anti-HLA-I antibody) and 4.49-fold (1 µg/mL of anti-HLA-I antibody) after antibody stimulation (**Figure 1D**).

The effect of diminished HO-1 expression in EC on monocyte adhesion and transmigration was analyzed after experimentally inducing HO-1 suppression. HO-1 knockdown was engendered by siRNA transfection and its efficiency was validated on mRNA as well as on the protein level. Twenty-four hours after transfection, HO-1 mRNA was already significantly reduced by 90% when compared to the nt-RNA-transfected control cells and reached its lowest expression after 48 h (**Supplementary Figure S1A**). Subsequently, a significant decrease in HO-1 protein concentration was seen 36 h after transfection and remained stable for up to 48 h (**Supplementary Figures S1B, C**).

After HO-1 knockdown in ECs, anti-HLA-I antibody stimulation resulted in a significant increase in adhesion and transmigration of monocytes compared to antibody-stimulated cells transfected with non-target (nt)-RNA. Adhesion was reinforced by 133% and transmigration by 214% (**Figures 1E, F**). Within the HO-1 siRNA-transfected groups, adhesion and transmigration were still significantly increased by anti-HLA-I antibody treatment when compared to the IgG2a antibody-treated ECs. As expected, a similar effect was observed in the groups transfected with nt-RNA-treated cells. Interestingly, HO-1 knockdown, when compared to nt-RNA-transfected ECs, also significantly increased transmigration in IgG2a-stimulated cells by 108%, suggesting that sufficient HO-1 expression in ECs is essential to maintaining EC homeostasis.

The effect of anti-HLA-I antibodies in the presence of induced HO-1 activity was further quantified. ECs were incubated with the HO-1 activator CoPPIX or statins that stimulate HO-1 as one of their pleiotropic effects, to increase HO-1 activity for 24 h prior to anti-HLA-I antibody stimulation. Compared to antibody stimulation alone, adhesion and transmigration were significantly decreased in CoPPIX-treated ECs to 42% and 84%, respectively (**Figures 1G, H**). Statin pretreatment of ECs decreased adhesion to 34% and transmigration to 77%. It can thus be concluded that stimulation of ECs with anti-HLA-I antibodies leads to increased numbers of adherent and transmigrated monocytes in a dose-dependent manner. Furthermore, genetic or pharmacological suppression of HO-1 concentration in EC intensifies anti-HLA-I antibody-driven effects. On the other hand, increasing HO-1 activity with CoPPIX or treatment with statins ameliorates anti-HLA-I antibody effects on adhesion and transmigration.

### 3.2 Anti-HLA-I antibody-mediated monocyte adhesion and transmigration depend on CD62E

**Figure 2:**
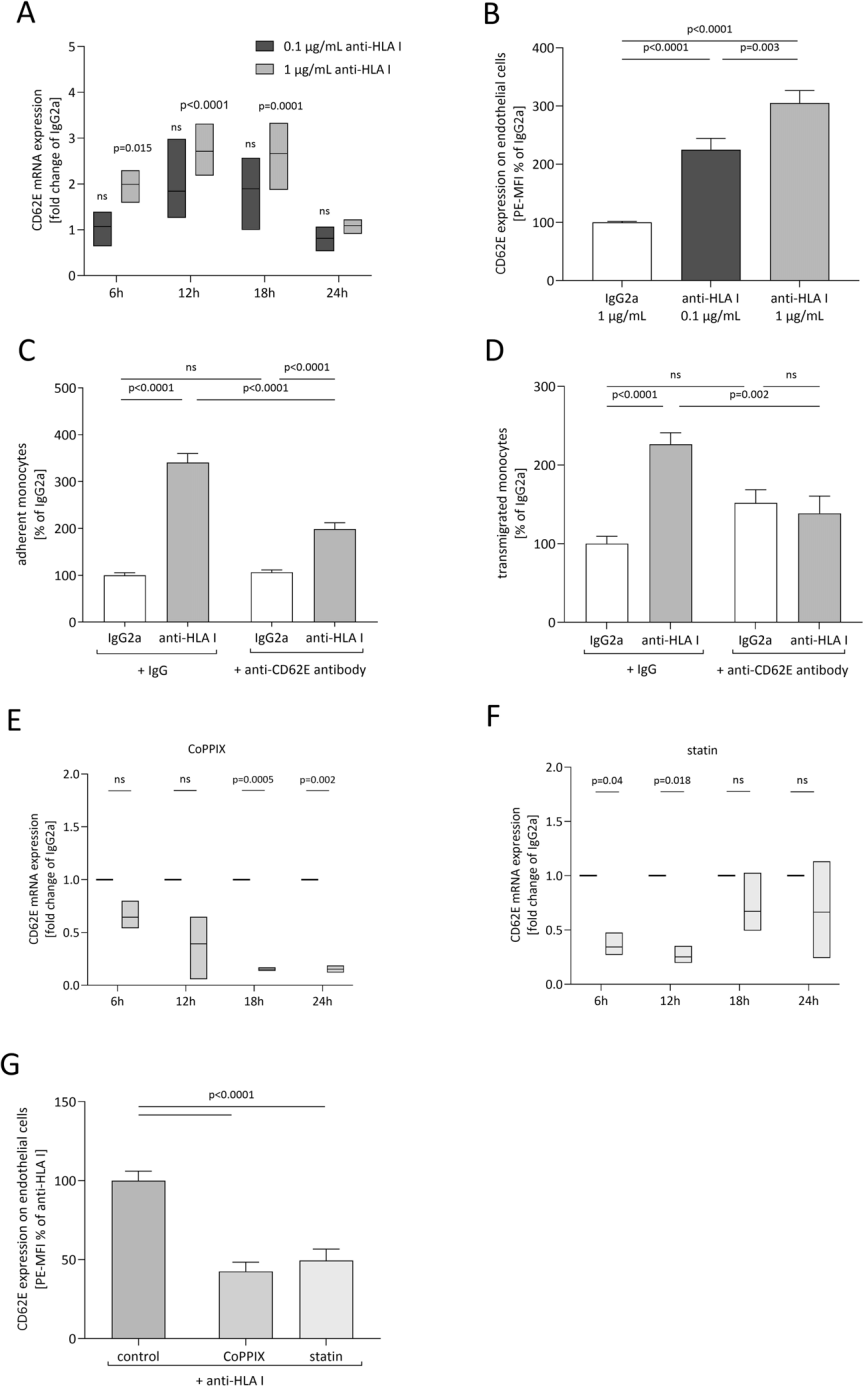
CD62E expression depends on anti-HLA-I stimulation and HO-1 activity, while its blockade decreases adhesion and transmigration. ECs were stimulated with 0.1 µg/mL (dark gray) or 1 µg/mL (light gray) of anti-HLA-I antibodies for 24 h in a starvation medium, and the expression of CD62E was analyzed. **(A)** At the indicated timepoints, fold change of CD62E mRNA level was measured and normalized to IgG2a (not shown)-treated ECs. **(B)** Mean fluorescence intensity (MFI) of PE-coupled anti-CD62E antibodies was measured to analyze the surface expression of CD62E on ECs after anti-HLA-I antibody stimulation. Data were normalized to IgG2a-treated ECs. **(C, D)** After stimulation with 1 µg/mL anti-HLA-I antibodies or IgG2a, ECs were incubated with anti-CD62E antibody or IgG isotype control for receptor blocking before adhesion or transmigration of THP-1 was quantified. **(E, F)** ECs were incubated with CoPPIX **(E)** or statins **(F)** for 24 h to induce HO-1 activity. After incubation, ECs were stimulated with 1 µg/mL of anti-HLA-I antibodies or IgG2a for up to 24 h in the presence of the corresponding HO-1 inducer. At the indicated timepoints, fold change of CD62E mRNA was measured using qPCR and normalized to IgG2a-treated ECs. **(G)** MFI of PE-coupled anti-CD62E antibodies was measured to analyze surface expression of CD62E on ECs after CoPPIX and statin incubation followed by 24 h of anti-HLA-I antibody stimulation. Data were normalized to anti-HLA-I antibody-treated ECs. Normally distributed data were analyzed using one-way (**B–D**, *n* = 9; **G**) or two-way (**A, E, F**, *n* = 3) ANOVA followed by Sidak’s multiple comparison test to adjust for multiple testing; statistically significant differences were assumed if *p <*0.05. ns, not significant.

To investigate the underlying mechanism of anti-HLA-I antibody-mediated adhesion and transmigration in more detail, ECs were screened for anti-HLA-I antibody-mediated changes of expression in a variety of established adhesion receptors. ECs were stimulated with two different antibody concentrations, and the transcriptome was collected and analyzed at indicated timepoints. At least a two-fold change of mRNA levels compared to the IgG2a control was considered a relevant change in expression. As confirmed by qPCR analysis, only mRNA levels for CD62E (E-selectin) fulfilled this criterion. A significant increase in CD62E mRNA concentration after 6, 12, and 18 h of anti-HLA-I antibody stimulation was observed (**Figure 2A**). In contrast, a two-fold mRNA increase was not obtained for JAM1, CD99, JAM3, ICAM2, or P-selectin (**Supplementary Figure S2**). Therefore, CD62E was the only EC adhesion receptor under investigation with our predefined cutoff value that proved susceptible to anti-HLA-I antibody stimulation. In addition, flow cytometry was used to clarify whether changes in mRNA expression resulted in alterations of CD62E expression on the surface of ECs (**Figure 2B**). Staining of EC with PE-coupled anti-CD62E antibodies following anti-HLA-I antibody stimulation showed a significant increase in CD62E protein concentration on the cell surface by 1.25-fold for 0.1 µg/mL of anti-HLA-I and by 2.05-fold for 1 µg/mL of anti-HLA-I. For CD62E surface expression, an anti-HLA-I concentration-dependent effect was not detected. To confirm the causal contribution of CD62E expression on the observed adhesion and transmigration results, ECs were incubated with 5 µg/mL of anti-CD62E antibody for 1 h after antibody stimulation. Blocking CD62E resulted in significantly reduced numbers of adherent and transmigrated monocytes compared to anti-HLA-I-activated cells incubated with an IgG control matching the CD62E blocking antibody. Adhesion decreased by 142% and transmigration by 88% (**Figures 2C, D**). Incubation with anti-CD62E isotype control IgG did not diminish anti-HLA-I-induced EC–monocyte interactions.

### 3.3 Target induction of HO-1 activity reduces anti-HLA-I induced CD62E expression

To further characterize the effects of CoPPIX- or statin-mediated HO-1 modulation on adhesion and transmigration, CD62E expression after increased HO-1 activity was quantified. mRNA expression was analyzed after 24 h of stimulation with HO-1 modulators followed by indicated time periods of anti-HLA-I stimulation in the presence of the respective HO-1 modulator. While anti-HLA-I antibody ligation alone significantly increased CD62E mRNA expression compared to IgG2a control antibody (as seen in **Figure 2A**), EC preincubation with CoPPIX or statins diminished these effects. In CoPPIX-treated ECs, a significant reduction of CD62E expression was observed after 18 and 24 h (**Figure 2E**), whereas statin-treated ECs showed a significant reduction after 6 and 12 h of anti-HLA-I antibody stimulation (**Figure 2F**). Alteration of CD62E protein expression on the surface of ECs was measured with flow cytometry, and a significant reduction of mean fluorescence intensity (MFI) of PE on CoPPIX- as well as statin-treated cells could be seen after 24 h (**Figure 2G**). In summary, CD62E expression is sensitive toward anti-HLA-I antibody ligation, and its expression can be modulated by HO-1 activity. Experiments with CD62E blocking antibodies revealed that the observed effects of anti-HLA-I antibody-induced adhesion and transmigration of monocytes are partially mediated by binding this specific surface receptor.

### 3.4 Experimental setup of heterotopic aortic transplantation model in mice to investigate the effects of *in-vivo* HO-1 modulation on TV

**Figure 3:**
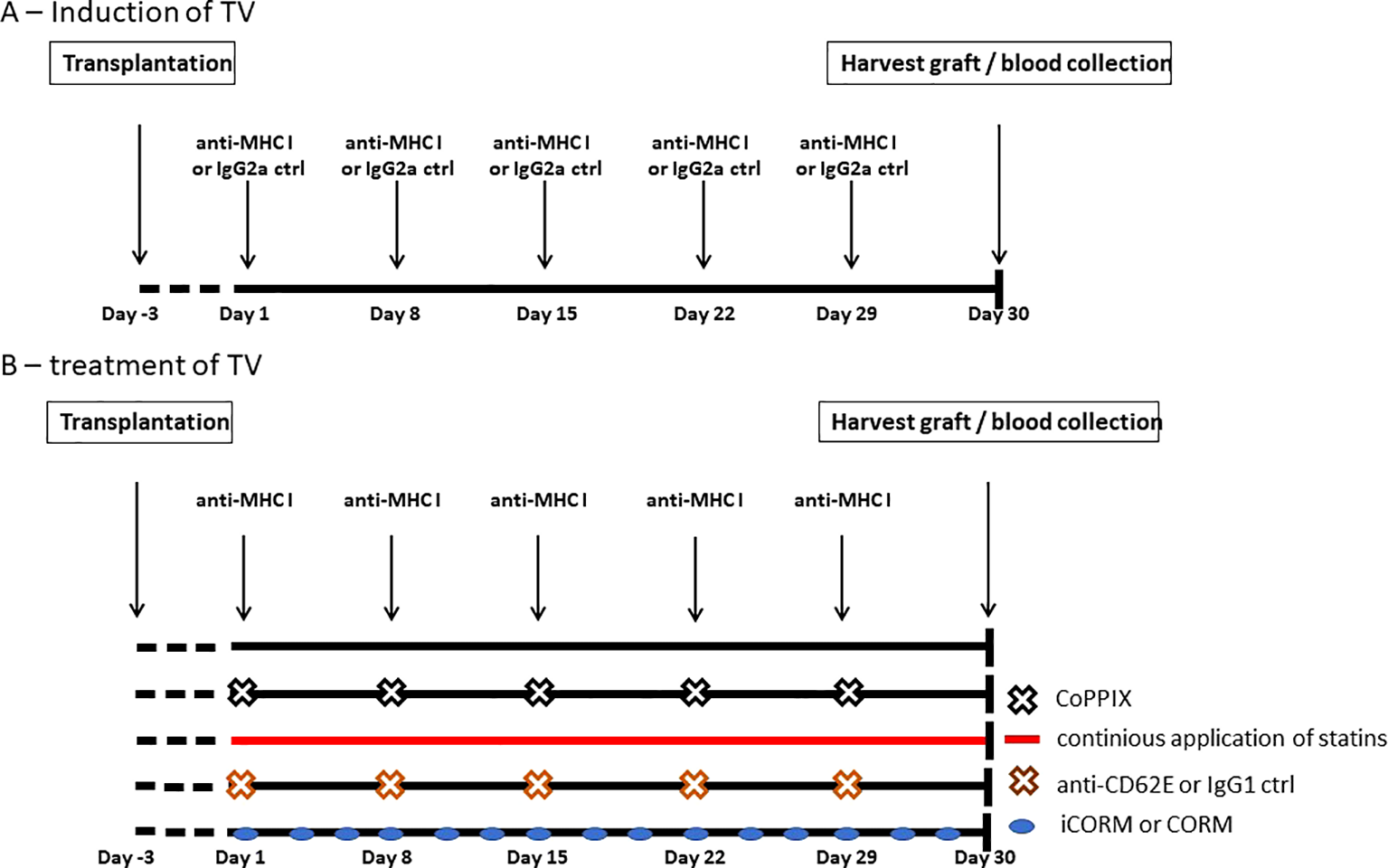
Experimental setup of antibody-induced and HO-1-mediated treatment of TV in Rag2 KO mice. Segments of thoracic aorta from Balb/c mice were transplanted into the abdominal aorta of Rag2 knockout (KO) mice. Experiments were begun on postoperative day 3. **(A)** Schematic drawing of DSA-induced TV. Mice received i.p. injections of anti-MHC I antibodies or IgG2a isotype control weekly to induce TV in the graft. After 30 days, mice were euthanized and the grafts as well as blood were collected. **(B)** Schematic drawing of the experimental setup for testing different compounds for reducing TV burden, which were applied in addition to anti-MHC I antibodies. CoPPIX was injected i.p. weekly and statins were continuously administered with the animals’ drinking water. CO-releasing molecule (CORM) or its inactive form (iCORM) was administered enterally, every other day. The CD62E blocking antibody or IgG1 isotype control was injected i.p. biweekly.

To establish an *in-vivo* model for investigating TV progression, aortic segments from Balb/c mice were transplanted infrarenally into the abdominal aorta of Rag2 KO mice. Combining mouse strains with different genetic backgrounds represents a full MHC mismatch model, and the application of Balb/c matching anti-MHC I antibodies evokes TV in grafts (30). Beginning on postoperative day (POD) 3, anti-MHC I antibody (SF1-1.1) or IgG2a isotype controls were injected weekly (**Figure 3A**). After 30 days, the mice were euthanized to collect the graft and blood. During the experiment, mice received either weekly intraperitoneal CoPPIX or statins administered continuously via drinking water for HO-1 modulation. To test the effects of the HO-1 downstream metabolite carbon monoxide (CO) on TV development, CORM or its inactive form (iCORM) was applied three times a week. For blocking experiments, anti-CD62E blocking antibodies were injected twice a week (**Figure 3B**).

### 3.5 HO-1 modulation minimizes monocytic infiltrates within the vessel wall of anti-MHC I-treated mice

**Figure 4:**
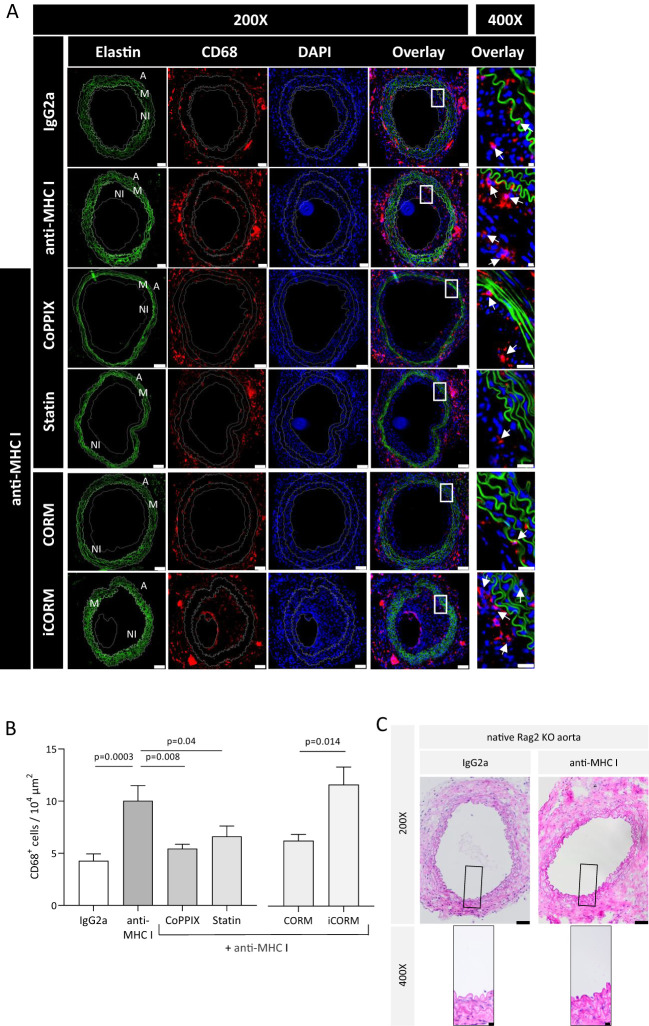
HO-1 modulation diminishes the monocytic infiltrate within the vessel wall of grafts. Thirty days after transplantation, Rag2 KO mice were euthanized, and cross-sections of the explanted grafts for immunohistochemical analysis were prepared. CD68 staining was performed every 75 µm of the graft to quantify the monocytic infiltrate. **(A)** Representative immunofluorescence images of CD68^+^ macrophages within the vessel wall (A, adventitia; M, media; NI, neointima). Elastic fibers (green) that made up the media of the vessel wall were pictured using autofluorescence; CD68 (red), DAPI (blue) (scale bar = 50 µm). High-magnification images of the box-indicated regions are shown for all treatment groups (scale bar = 5 µm). Arrows indicate macrophages. **(B)** Statistical analysis of CD68^+^ macrophages in the vessel wall. The number of CD68^+^ macrophages was statistically evaluated using a one-way ANOVA. Parametric data are shown as mean ± SEM and were adjusted for multiple testing; statistically significant differences were assumed if *p <*0.05. **(C)** Representative H&E images of Rag2 KO aorta at ×200 (scale bar = 50 µm) and indicated areas at ×400 magnification (scale bar = 10 µm). Grafted Rag2 KO mice were administered with anti-MHC I antibody or IgG2a to induce TV. Graft and native Rag2KO aorta were explanted after 30 days and analyzed. Here, segments of the native Rag2 KO (not the graft) aorta of mice from both groups are shown.

According to *in-vitro* data where anti-HLA-I antibodies support the adhesion and transmigration of mononuclear cells, one may conclude that monocytes are a driving force in TV development. To prove this assumption, the extent of monocytic infiltrate within the graft was analyzed to investigate whether applying anti-MHC I antibodies promotes monocyte infiltration *in vivo*, as well as if HO-1 modulation has a preventive effect on monocyte trafficking. Grafts were harvested after 30 days and serial cross-sections were stained for CD68^+^ monocytes. In addition, the autofluorescence of elastin fibers indicating medial structures was recorded. Grafts from anti-MHC I-treated mice harbor significantly more CD68^+^ cells in their vessel walls than those of isotype control-treated animals (**Figure 4A**). On average, the number of CD68^+^ cells in control mice was 4.2 × 10^−4^ µm^2^ and thus significantly lower compared to 10 × 10^−4^ µm^2^ CD68^+^ cells in anti-MHC I-treated mice. In accordance with our *in-vitro* findings, weekly injections of CoPPIX reduced the number of monocytes significantly to 5.4 × 10^−4^ µm^2^, and statin administration lowered the number of CD68^+^ cells to 6.6 × 10^−4^ µm^2^. Analysis of CORM-treated mice revealed a significantly lower number of CD68^+^ cells compared to the iCORM control group (**Figure 4B**). Weight gain, documented once a week for general health monitoring, did not differ over time between the experimental groups (**Supplementary Figure S3**). Furthermore, the hematoxylin and eosin (H&E)-stained segments of Rag2 KO aortas did not show TV or signs of inflammation in the Rag2 KO vasculature, indicating that anti-MHC I antibodies did not induce unspecific vascular inflammation outside of the graft (**Figure 4C**). Our CD68 staining proves that administering anti-MHC I antibodies is sufficient to induce the formation of a monocytic infiltrate in transplanted aortic segments in the absence of an adaptive immune response. Targeted HO-1 induction attenuates the number of monocytes within the vessel wall, subsequently reducing neointima formation and, ultimately, the burden of TV. Similarly, the HO-1 metabolite CO leads to a reduction of monocyte numbers regardless of its solvent DMSO. H&E staining of the native Rag2 KO aorta does not show TV, demonstrating that DSAs (anti-H2-K^d^) specifically affect the graft’s vasculature.

### 3.6 HO-1 modulation reduces anti-MHC I antibody-mediated TV burden in grafts

**Figure 5:**
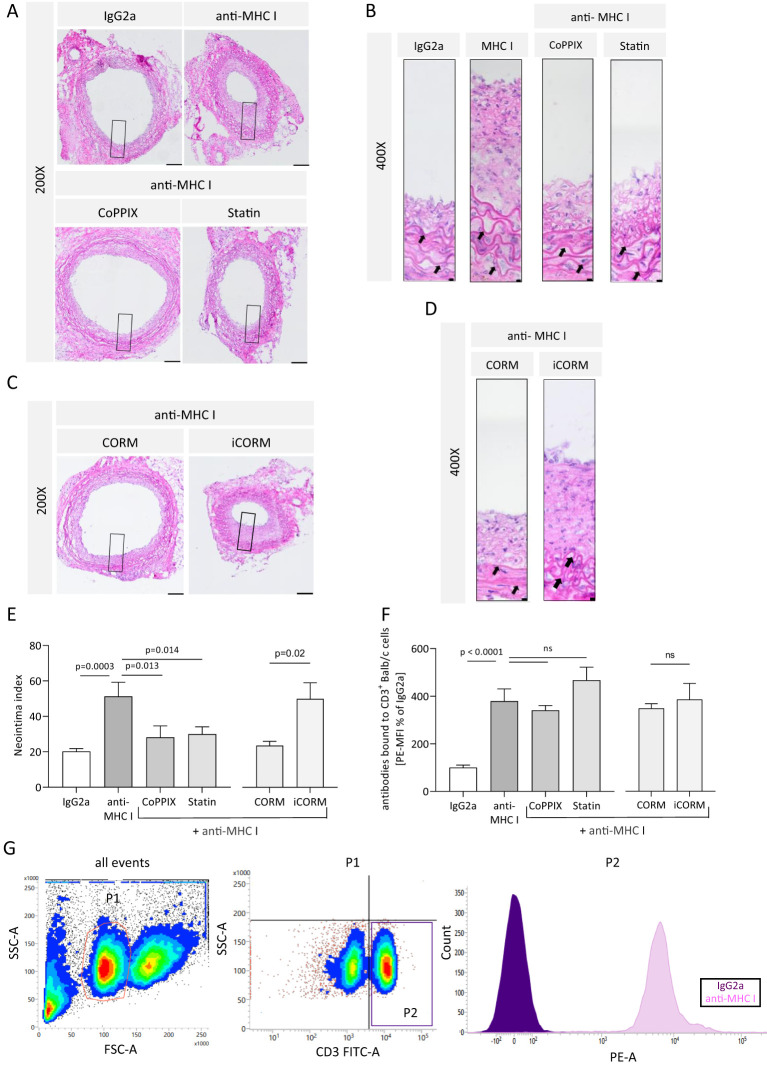
HO-1 modulation mitigates anti-MHC I antibody-induced neointima hyperplasia *in vivo*. The neointima index was derived from H&E-stained sections every 75 µm of the graft. **(A, C)** Representative images of different treatment groups at ×200 magnification (scale bar = 100 µm). High-magnification images of the box-indicated regions are shown in **(B)** and **(D)** (scale bar = 5 µm). Arrows indicate elastin fibers of the media. **(E)** Statistical analysis of the neointima index of *n* = 6–8 mice per group. **(F)** Mean fluorescence intensity (MFI) of PE on CD3^+^ cells was determined and normalized to the plasma of IgG2a-treated control mice. Normally distributed data (mean ± SEM) were analyzed using a one-way ANOVA or two-way ANOVA **(F)** followed by Sidak’s multiple comparison test to adjust for multiple testing; statistically significant differences were assumed if *p <*0.05. **(G)** Gating strategy to confirm the presence of anti-MHC I antibodies within the circulation of Rag2 KO mice. Balb/c splenocytes were incubated with plasma from recipient mice and stained with a combination of anti-CD3 antibodies coupled with FITC and PE-coupled anti-mouse IgG. CD3^+^ cells were gated as seen in the representative scatterplot. ns, not significant.

We analyzed the extent of neointima formation to evaluate whether reduced monocytic infiltrate within the vessel wall correlates with reduced pathophysiological TV manifestation. Serial cross-sections were stained with H&E to quantify the extent of anti-MHC I-induced neointima hyperplasia. Administration of antibodies once a week resulted in extensive neointima formation compared to IgG2a-treated mice. Otherwise, pharmacologically induced HO-1 activity with CoPPIX or statins prevented anti-MHC I antibody-induced neointima formation (**Figures 5A, B**). The media of the vessel wall was identified by elastin and collagen fibers, which are arranged concentrically within it (**Figure 5B**, arrows). The effects of the HO-1 downstream metabolite CO on antibody-induced neointima formation were investigated in mice treated with either iCORM or CORM. Grafts of mice treated with anti-MHC I antibodies and CORM revealed less newly formed neointima when compared to mice who received iCORM (**Figures 5C, D**). For statistical analysis, the NI was calculated and compared between the treatment groups. Mice treated with anti-MHC I antibodies showed a significantly higher NI of 51.27 in comparison to IgG2a control animals, with an NI of 20.23 (**Figure 5E**). CoPPIX treatment to induce HO-1 limited the NI to 28.3 and continuously applied statins significantly lowered it to 29.97. Additionally, the HO-1 metabolite CO released from CORMs also led to a significant reduction in the calculated NI to 23.6, compared to its iCORM control with an NI of 50.

The presence of anti-MHC I antibodies within the circulation of recipient mice was confirmed using flow cytometry. CD3^+^ Balb/c splenocytes were incubated with the plasma of Rag2 KO mice collected at the end of the experiment and identified with FITC-coupled anti-CD3 antibodies. Bound anti-MHC I antibodies from the plasma on CD3^+^ splenocytes were determined using PE-coupled anti-IgG antibodies and quantified by the MFI of PE. The results revealed a significant increase in bound anti-MHC I antibodies to Balb/c CD3^+^ splenocytes incubated with plasma samples from antibody-treated mice when compared to the control mice. Plasma from CoPPIX-, statin-, CORM-, and iCORM-treated mice, in addition to passively transferred antibodies, showed no significant decrease in bound antibodies compared to the antibody-treated group, thus proving that the observed effects of minimized NI are not dependent on lower antibody concentrations (**Figure 5F**). The corresponding gating strategy is shown in **Figure 5G**. Taken together, we were able to successfully induce TV in transplanted aortic segments between genetically different mice strains. Targeted induction of HO-1 activity as well as the administration of the HO-1 downstream metabolite CO resulted in alleviated neointima formation. This finding suggests that the HO-1 pathway plays a causal role in minimizing proinflammatory conditions. Furthermore, we were able to detect circulating anti-MHC I antibodies in the blood of all mice who received this antibody.

### 3.7 CD62E expression on vascular smooth muscle cells is intensified in grafts after anti-MHC I treatment

**Figure 6:**
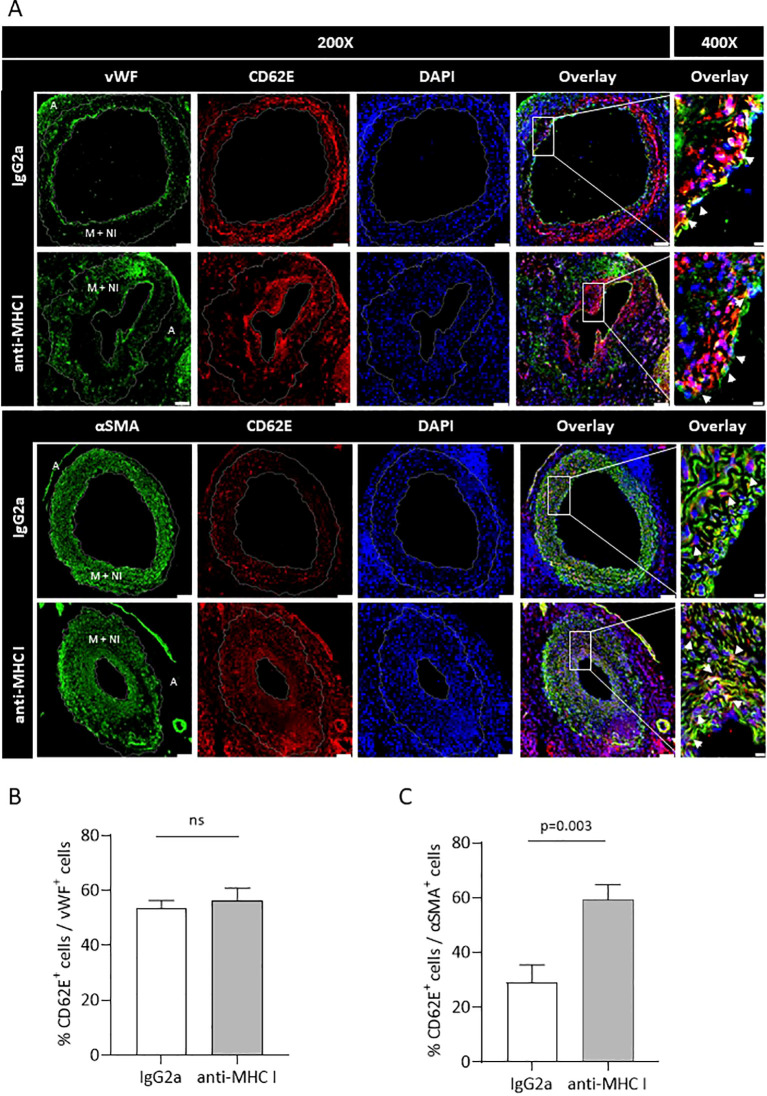
Anti-MHC I antibody induces CD62E expression on VSMCs but not on ECs. Serial cross-sections of grafts after 30 days of anti-MHC I antibody injection were double-stained for either CD62E/EC or CD62E/VSMC to quantify CD62E expression on different cell types of the graft’s vessel wall. **(A)** Immunofluorescence micrographs of MHC I antibody- or IgG2a-treated mice for ECs (vWF, green) VSMC (αSMA, green), CD62E (red), and DAPI (blue) at ×200 magnification (scale bar = 50 µm). Box-indicated areas are enlarged at ×400 magnification (scale bar = 5 µm), and physiologically different parts of the vessel wall are indicated on vWF- and αSMA-stained sections (A = adventitia, M + NI = media + neointima). **(B)** Statistical analysis of the relative number of CD62E^+^ ECs and **(C)** relative number of CD62E^+^ VSMCs. Normally distributed data are shown as mean ± SEM and were analyzed using Student’s *t*-test; statistically significant differences were assumed if *p <*0.05; *n* = 8 mice per group. ns, not significant.

Serial cross-sections of grafts of anti-MHC I- or IgG2a-treated mice were double-stained for either CD62E and EC marker von Willebrand factor (vWF) or CD62E and VSMC marker αSMA to quantify the influence of antibody application on CD62E expression on ECs and VSMCs *in vivo*. The relative amounts of CD62E^+^ ECs and VSMCs were measured (**Figure 6A**). Weekly applications of anti-MHC I antibodies did not induce CD62E expression on graft ECs compared to those of the control mice. In both groups, approximately 55% of ECs were stained positive for CD62E (**Figure 6B**). Nevertheless, immunostaining revealed a profound area of CD62E-positive cells within the vessel wall. In the grafts of IgG2a isotype control mice, 29% of VSMCs stained positive for CD62E; 59% of VSMCs, however, expressed CD62E in the anti-MHC I antibody group (**Figure 6C**). VSMC rather than EC cells were mainly responsible for CD62E expression in graft vasculature after 30 days of DSA treatment.

### 3.8 Application of anti-CD62E antibody diminished anti-MHC I-mediated TV

**Figure 7:**
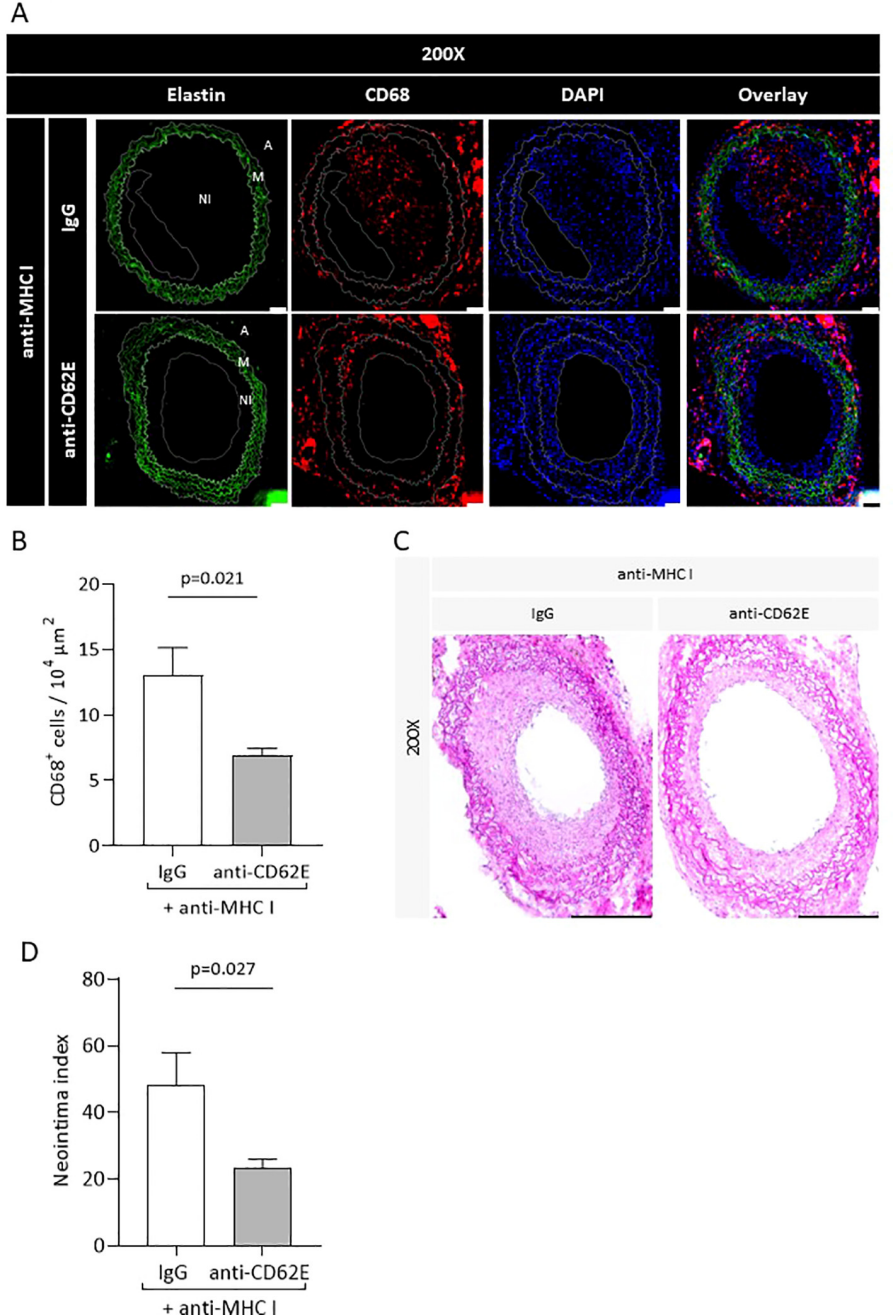
Blockade of CD62E results in reduced monocyte numbers in the vessel wall and attenuates the neointima index. Transplanted mice received CD62E blocking antibody or isotype control **(IgG)** biweekly in addition to anti-MHC I antibody administration. After 30 days, the grafts were harvested and histologically analyzed. **(A)** Immunohistological staining for elastin (autofluorescence, green), CD68 (red), and DAPI (blue) to quantify CD68^+^ monocytes (scale bar = 50 µm). **(B)** Statistical analysis of CD68^+^ monocytes. **(C)** Images of H&E stainings of mice who received a combination of anti-MHCI antibody and CD62E antibody or IgG (scale bar = 50 µm). **(D)** Statistical analysis of neointima index between groups.

The *in-vitro* experiments demonstrated that the application of an anti-CD62E blocking antibody on antibody-stimulated EC causes reduced monocytic adhesion as well as transmigration. We therefore tested the effects of CD62E blockade in our *in-vivo* mouse model. Mice were injected with DSA once a week to induce TV. They then received an additional anti-CD62E antibody or the corresponding isotype control twice a week. Grafts were harvested after 30 days and immunohistochemically processed for CD68 and H&E staining to quantify the extent of the monocytic infiltrate within the vessel wall as well as the newly formed neointima. Immunofluorescence evaluation revealed that the grafts of mice treated with anti-CD62E antibodies bear significantly less CD68^+^ monocytes within the vessel wall than those of control animals (**Figures 7A, B**). The intima index, calculated on chromogen-stained serial cross-sections, significantly decreased from 48.09 in isotype-treated mice to 23.25 in CD62E blocking antibody-treated mice (**Figures 7C, D**). There were no differences in weight fluctuations between the groups (**Supplementary Figure S4**). In accordance with prior *in-vitro* experiments, we proved that CD62E blockade in an allogenic transplantation model is sufficient for reducing the number of monocytes within the vessel wall and lowering the extent of neointima formation.

### 3.9 Anti-MHC I antibodies induce profound changes of soluble cytokine expression in mice

**Figure 8:**
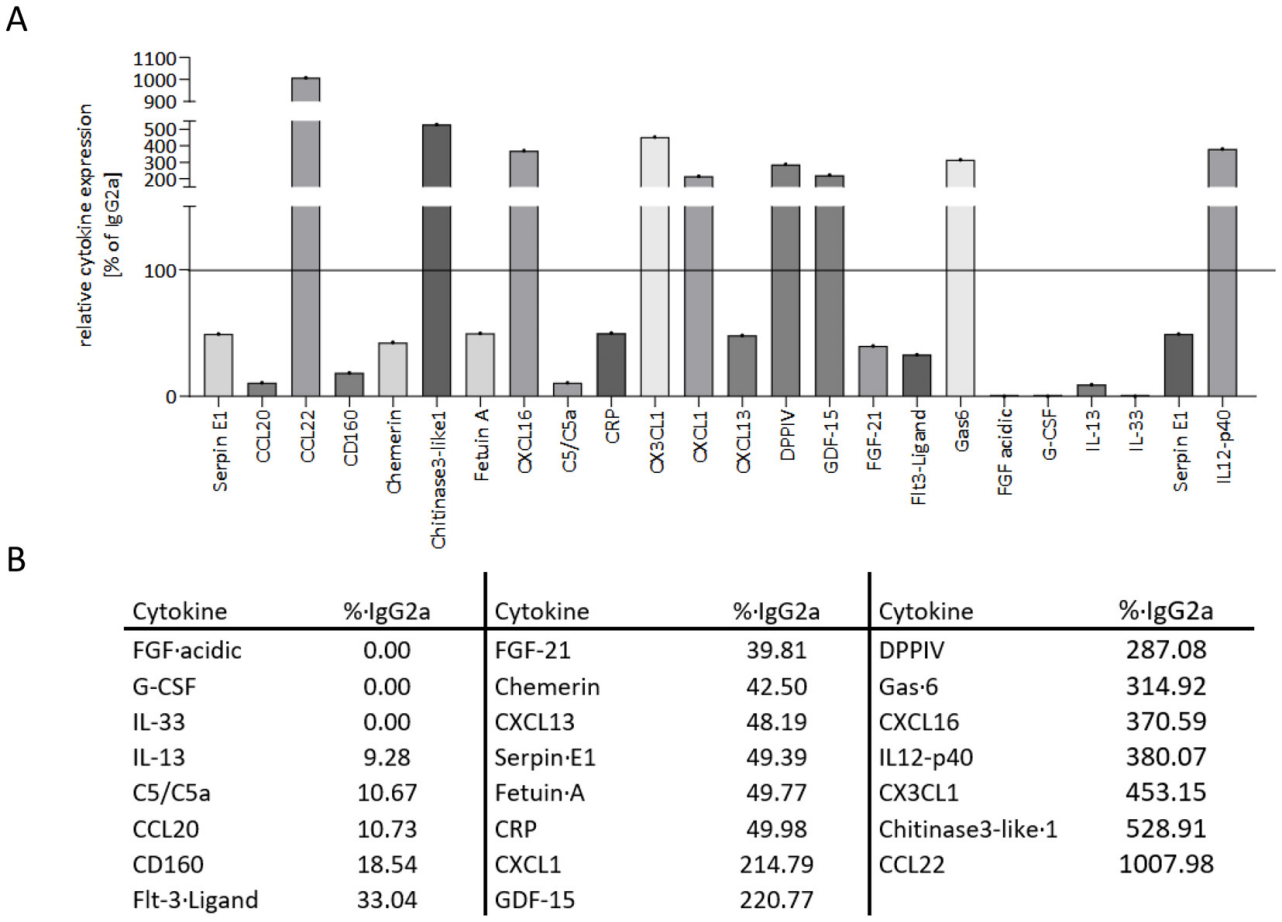
Anti-MHC I antibody induces up- and downregulation of different cytokines in mice. Plasma from transplanted mice was collected after 30 days of anti-MHC I antibody or IgG2a stimulation. With the membrane-based cytokine array, the expression profile of 80 cytokines was determined and compared to IgG2a isotype control. **(A)** Descriptive presentation of 23 cytokines that revealed a physiologically relevant change. **(B)** Table of the cytokines from **(A)** with their corresponding expression normalized to IgG2a.

Plasma was analyzed to semiquantify the expression of different cytokines after applying the anti-MHC I antibody and compared to the cytokine profile of control mice. We were able to measure 80 of 111 cytokines that could have been technically detected with the assay used. A reduction of more than 50% or an increase of more than two-fold compared to the control plasma was seen as physiologically relevant. For the sake of clarity, only these 23 physiologically relevant cytokines are shown in **Figure 8A**. The cytokine CCL22 was subject to the greatest change with a 10-fold increase of expression compared to the expression in control plasma. CXCL1, GDF-15, and DPPIV were more than twice as high as the control, and Gas-6, CXCL16, IL12-p40, CX3CL1, and Chitinase3-like1 were increased by a factor of 3 to 5 (**Figure 8B**). The remaining cytokines in the table were clearly decreased or no longer detectable at all in the plasma of antibody-treated mice.

In the cell culture supernatants, we measured 36 of 105 technically detectable cytokines of which only two had physiologically relevant changes (**Supplementary Figures S5A, B**).

## 4 Discussion

The main obstacle to long-term graft survival after solid organ transplantation is DSA-evoked TV. DSAs bind endothelial cells of the vasculature and induce activation of this specific cell population (43). Activation with proinflammatory stimuli results in endothelial dysfunction, further leading to expression changes within adhesion receptors on the surface, subsequent trafficking of immune cells, and chronic inflammation (44). In the context of chronically inflamed vessel walls, activation of different anti-inflammatory pathways, such as the HO-1 pathway, leads to accommodation, a condition in which DSAs do not further harm the graft (45). Here, we demonstrated *in vitro* and in a murine allogenic aortic transplantation model that stimulation and activation of human primary artery endothelial cells with anti-HLA-I antibodies reinforces CD62E-dependent interactions between ECs and monocytes. Furthermore, we provided evidence that targeted induction of HO-1 diminishes these effects and protects against the development of transplant vasculopathy.

Besides non-immunological factors such as ischemia–reperfusion injury (46), mode of brain death (47), and cold ischemia time (48), several immunological factors initiate EC activation and are reflected in the criteria for chronic antibody-mediated rejection. Binding of DSA on EC induces activation of the complement system, as seen in C4d-positive staining of graft biopsies (44). The presence of DSA and C4d covalently bound to EC does not necessarily lead to pathological graft changes (13). Host-derived immune cell infiltrates within the vessel wall consist predominantly of T cells, macrophages, NK cells, and to a lesser extent, dendritic cells (49). How each of these cell types contributes to TV development, as well as how they influence each other, remains under investigation. Stimulating human EC with anti-HLA-I antibodies induced a concentration-dependent increase in adhesion and transmigration of monocytes as typically seen with other proinflammatory stimuli (50–52). Furthermore, anti-HLA-I antibody stimulation results in an increased expression of the adhesion receptor CD62E on the surface of ECs. CD62E, as a member of the selectin family, provides the initial temporary interaction between ECs and monocytes and is responsible for extracting leukocytes from blood circulation. It is already known that CD62E expression is sensitive toward proinflammatory stimuli, such as TNF-α and LPS (53–55). In contrast to CD62P (P-selectin), which plays a major role in leukocyte homing, CD62E binding activates leukocytes via phosphorylation of the intracellular AKT and NF-κB pathways (56). Furthermore, CD62E binding via its corresponding ligands on leukocytes activates β_2_-integrins, which in turn results in enhanced transmigration (57). The regulation of CD62E by anti-HLA antibodies, however, has not been shown before. Furthermore, our CD62E blocking experiments have proven the functional relevance of anti-HLA-I-mediated CD62E expression on ECs. Blocking CD62E and therefore reducing the number of available adhesion receptors for monocytes diminish the number of adherent as well as transmigrated immune cells. These observed effects have been intensified by the HO-1-specific siRNA transfection of EC. HO-1 catalyzes the rate-limiting step of heme degradation (58) and, beyond that, plays an important role in anti-inflammatory pathways in order to protect ECs from activation (59, 60). Successfully reduced HO-1 protein expression has a noticeable effect on increased adhesion and monocyte transmigration. Our studies indicate that target induction of HO-1 with CoPPIX as well as incubation with statins results in decreased anti-HLA-I-mediated CD62E expression and minimized adhesion and transmigration of monocytes after anti-HLA-I ligation to ECs. Both reagents induce HO-1 expression and prevent EC activation (37, 61). It is also worth noting that statins exert a plurality of pleiotropic effects independent of the HO-1 pathway. Statin treatment mitigates NF-κB pathway activation, which itself mediates DSA-induced EC activation (62, 63). Nevertheless, sufficient HO-1 expression is mandatory for maintaining EC homeostasis, and its deficiency results in serious chronic inflammation and a shortened life expectancy in mammals (64, 65).

Chronic inflammation of the endothelium due to bound DSAs results in neointima formation and monocytic infiltrate within the vessel wall (44, 66). Both features are hallmarks of chronic transplant rejection, or so-called transplant vasculopathy. For our present study, we adapted the allogenic transplantation mouse model established by Koulack et al. (41) and successfully evoked TV by utilizing anti-MHC I antibodies. Mice treated with anti-MHC I antibodies revealed significantly increased neointima formation compared to isotype-treated control mice. Besides these local effects on the vessel wall, anti-MHC I antibodies induce profound changes in the plasma cytokine profile. The increased expression of soluble factors such as CCL22, CXCL16, CX3CL1, and CXCL1, which all play a crucial role as chemoattractants (67–70), proves the systemic effects of anti-MHC I antibodies in mice. In the experiments carried out here, it is not possible to distinguish whether these soluble factors have been released by ECs or already transmigrated immune cells, serving as a self-amplifying trigger. The HO-1 activators CoPPIX or statins lead to a reduced chronic inflammation of the vessel wall, which was manifested by a decreased neointima index. Upregulated expression of anti-inflammatory genes (such as Bcl-2, Bcl-xL, and HO-1) in the context of accommodation alleviates ICAM-1 and Il-1β expression and protects grafts from rejection (33). Furthermore, target upregulation of HO-1 impairs proinflammatory conditions and protects against viral infections, acute rejection, and cellular infiltration (71). Besides its beneficial effects on reduced neointima formation, CoPPIX and statins also diminish the extent of monocytes within the vessel wall. These observations are not associated with systemic inflammation. Mice treated with CO also benefit from a decreased TV burden. CO, a downstream metabolite within the HO-1 pathway, impedes TNF-α, IL-1, and MIP-1β (36). Beyond that, the application of CO after liver transplantation delays rejection (72). Transmigrated monocytes, whose numbers directly influence fibrosis in transplanted organs (73), are a driving force of neointima formation and therefore TV development. Growing concentrations of IL-12p40 in the periphery might be responsible for the induced recruitment of monocytes (74). Once recruited to the transplant, transmigration of monocytes into the vessel wall might be mediated by binding CD62E. Shortly after CD62E binding, leukocytes paracellularly transmigrate from the vasculature into the vessel wall, where they adopt proinflammatory phenotypes (27, 66). We have proven that VSMC of the graft increases CD62E expression on its surface and that CD62E blocking antibody ameliorates anti-MHC I-induced monocytic infiltrate and neointima formation.

Therapeutical options to treat TV or to decelerate its development are limited and there is an urgent need for improved therapies. Target induction of the anti-inflammatory enzyme HO-1 might be a possible solution, and we have shown that this goal can be achieved by statin therapy. Besides their LDL-lowering effects, statins induce HO-1 upregulation, prevent EC activation, and protect the cardiovascular system (75, 76). In our experiments, statins and CoPPIX were administered systemically to activate HO-1, but it also possible to overexpress HO-1 in the target tissue by viral transfection approaches.

## Data Availability

The original contributions presented in the study are included in the article/**Supplementary Material**. Further inquiries can be directed to the corresponding author.
