# Supplementary material for: Heme oxygenase-1 modulates CD62E-dependent endothelial cell–monocyte interactions and mitigates HLA-I-induced transplant vasculopathy in mice
Source: Front Immunol. 2025 Mar 7;16:1447319. doi: 10.3389/fimmu.2025.1447319 (PMC11925954; doi:10.3389/fimmu.2025.1447319)
Supplement: Supplementary file 1 [file DataSheet1.pdf]

Supplementary Material

1 Supplementary Figures

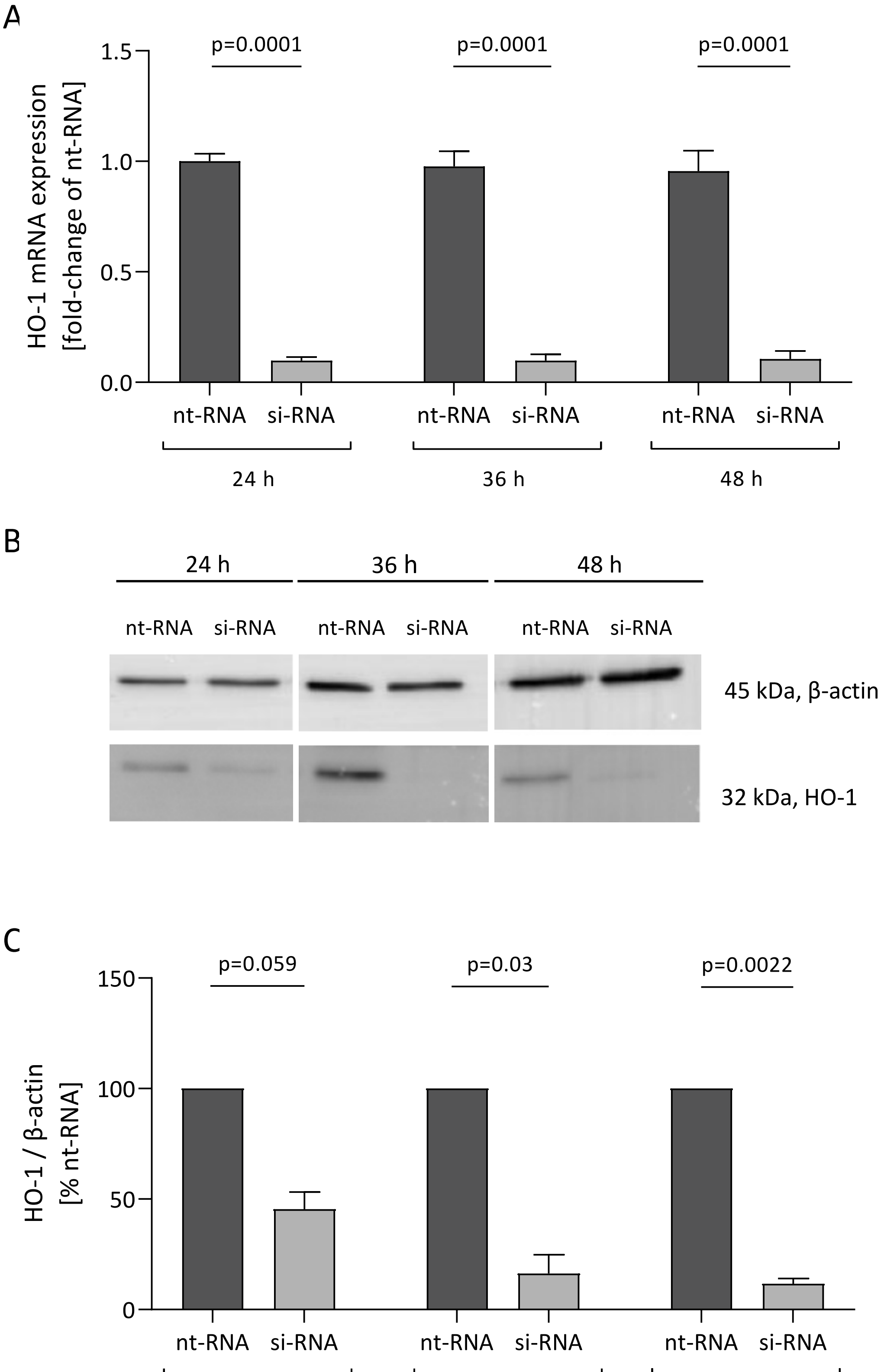

Figure 1 – Transfection of HO-1 specific siRNA reduces HO-1 expression in EC.

HCAEC were transfected with HO-1 specific si-RNA or non-target (nt)-RNA as control and. (A) At indicated time points (A) the transcriptome was collected and HO-1 mRNA expression were analyzed or (B) the proteome was collected to investigate the effects of HO-1 siRNA transfection on HO-1 protein expression. (C) statistical analysis of HO-1 Western blots, β-Actin was used as loading control. Statistically significant differences were assumed if $p < 0.05$; $n = 3$.

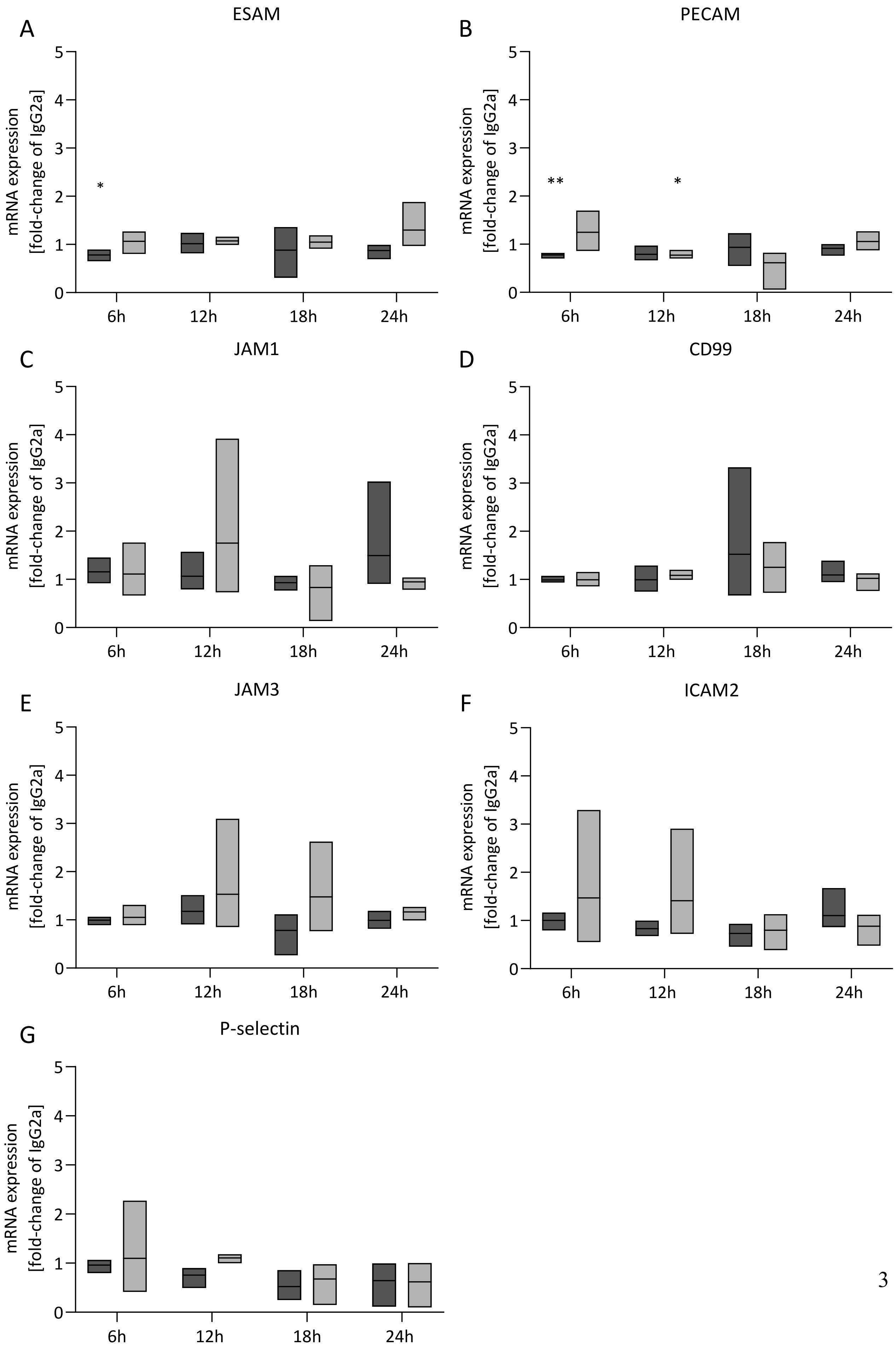
A
ESAM
B
PECAM
C
JAM1
D
CD99
E
JAM3
F
ICAM2
G
P-selectin
mRNA expression
[fold-change of IgG2a]
0
1
2
3
4
5
6h
12h
18h
24h
*
**
*

Figure 2 – Screening of various adhesion receptors

HCAEC were stimulated with 1 µg/ml anti-HLA I antibody or IgG2a isotype control. At indicated time points the transcriptome was collected and qPCR experiments were performed. The fold-change of mRNA expression of different adhesion receptors was calculated to IgG2a stimulated control cells. Normally distributed data were analyzed using two-way ANOVA followed by Sidak's multiple comparison test to adjust for multiple testing; statistically significant differences were assumed if $p < 0.05$; n = 3.

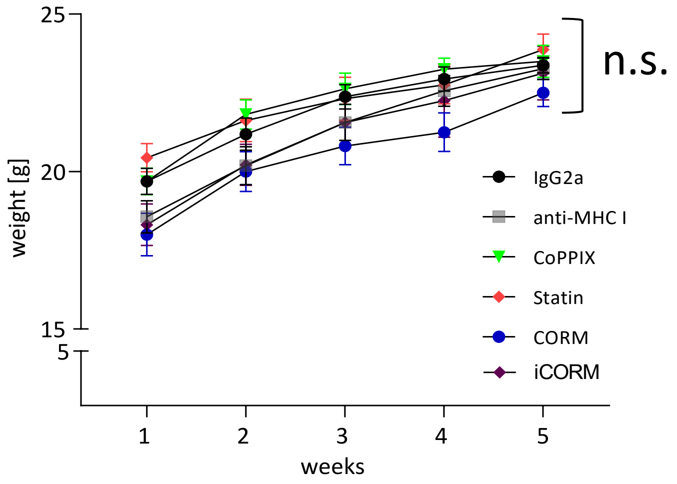

Figure 3 – Weight development of mice over time

Weight fluctuations among mice under the experimental conditions were monitored for health surveillance and to exclude systemic inflammation. Data were analyzed using a two-way ANOVA followed by Sidak's multiple comparison test to adjust for multiple testing. Statistically significant differences were assumed if $p < 0.05$; n = 6-8 mice per group.

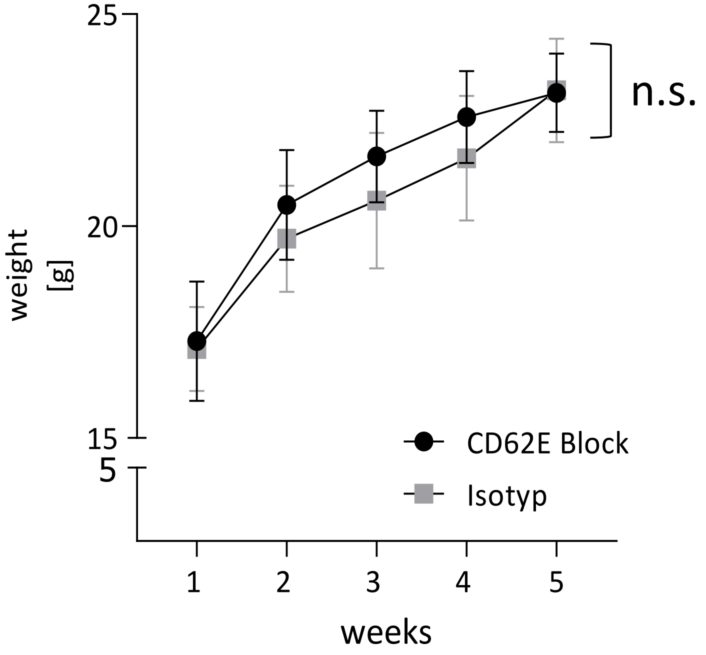

Figure 4 – Weight development of mice over time

Weight fluctuations of mice under experimental conditions was monitored for health surveillance and to exclude systemic inflammation. Normally distributed data were analyzed using two-way ANOVA followed by Sidak's multiple comparison test to adjust for multiple testing; statistically significant differences were assumed if $p < 0.05$; n = 5-6 mice per group.

Suppl. Fig. 5

A

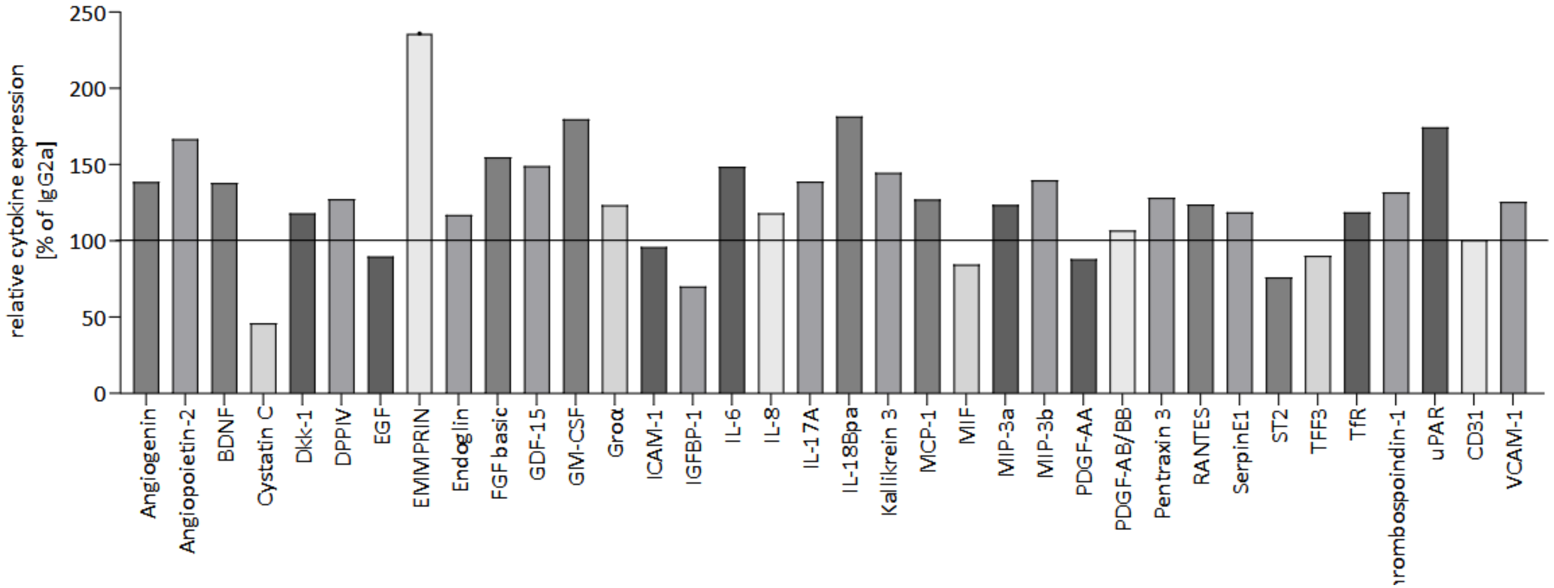

B

Cytokine	%·IgG2a	Cytokine	%·IgG2a	Cytokine	%·IgG2a
Cystatin·C	46.17	Dkk-1	118.17	Angiogenin	138.76
IGFBP-2	70.20	TfR	118.89	IL-17A	138.89
ST2	76.00	Serpin·E1	118.90	MIP-3β	139.65
MIF	84.63	Groα	123.41	Kallikrein·3	144.70
PDGF-AA	87.95	RANTES	123.57	IL-6	148.66
EGF	89.88	MIP-3a	123.66	GDF-15	.149.19
TFF·3	90.19	VCAM-1	125.68	FGF·basic	154.89
ICAM-1	95.87	MCP-1	127.21	Angiopoietin	166.73
CD31	100.79	DPPIV	127.39	µPAR	174.56
PDGF-AB/BB	106.85	Pentraxin·3	128.31	GM-CSF	179.84
Endoglin	116.88	Thrombospon.1	131.80	IL-18·Bpa	181.56
IL-8	118.14	BDNF	137.95	EMMPRIN	235.95

Confidential - Company Proprietary

Figure 5 – Anti-HLA I antibody only induces a few changes in cytokines in humans

Supernatants from EC were collected after 24 h stimulation either with anti-HLA I antibody or IgG2a. (A) Descriptive presentation of expression profile of 36 cytokines that were detected with a membrane-based array. Change in expression is compared to the expression if IgG2a stimulated EC. (B) Table of the cytokines from A with their corresponding expression normalized to IgG2a.

Confidential - Company Proprietary
